# Nuclear Factor of Activated T Cells (NFAT) Proteins as Targeted Molecules in Diseases: A Narrative Review

**DOI:** 10.7759/cureus.75844

**Published:** 2024-12-16

**Authors:** Mohadese Mozafari, Siti Nurnasihah Md Hashim, Khairul Bariah Ahmad Amin Noordin, Siti Aishah Zainal, Ahmad Azlina

**Affiliations:** 1 Basic and Medical Sciences Unit, School of Dental Sciences, Universiti Sains Malaysia Health Campus, Kubang Kerian, MYS; 2 Human Genome Centre, School of Medical Sciences, Universiti Sains Malaysia Health Campus, Kubang Kerian, MYS; 3 Tissue Bank Unit, School of Medical Sciences, Universiti Sains Malaysia Health Campus, Kubang Kerian, MYS

**Keywords:** angiogenesis, cancer, endothelial cell, nfat pathway, nfat proteins, stem cell

## Abstract

The nuclear factor of activated T cells (NFAT) is a key player in the NFAT pathway, regulating various cellular processes physiologically and pathologically. NFAT signaling is implicated in developing multiple diseases, such as cancer progression, that are associated with angiogenesis. Despite numerous studies on NFAT, there is still a dearth of information on the proteins and signaling pathway compared to other established pathways. With five NFAT proteins in the spotlight, this review aims to update the understanding of their roles and signaling by analyzing the most recent studies on the NFAT pathway. The recent insights into NFAT proteins and their association with diseases enhance our understanding of these proteins and open the possibility of developing therapeutic strategies for such diseases.

## Introduction and background

The nuclear factor of activated T cells (NFAT) pathway, particularly related to angiogenesis, is the primary focus of this review. This compilation of the latest research on the NFAT pathway underscores its profound significance in a wide range of physiological and pathological processes. Angiogenesis, the process of blood vessel formation, is a crucial area of interest due to its occurrence in embryonic development, ovulation, wound healing, inflammation, and cancer [1]. Understanding the role of the NFAT proteins and the corresponding signaling pathways in the development of these processes and diseases will underscore the importance of these proteins and their pathway for further investigation.

The NFAT family proteins were first discovered as transcription factors with multiple regulatory functions in nuclear extracts of activated T cells [2]. NFAT is expressed in T cells and other immune and non-immune cells throughout the vertebrate system. The NFAT family consists of five proteins, including NFATc1 (also known as NFAT2 or NFATc), NFATc2 (NFAT1 or NFATp), NFATc3 (NFAT4 or NFATx), and NFATc4 (NFAT3), which are all regulated by calcium/calcineurin [3], whereas NFAT5 is not [4].

NFAT signaling

There are two NFAT pathways: calcineurin-dependent and independent. The NFAT-dependent pathway is the most extensively studied. Changes in intracellular calcium, largely mediated by ligand-gated store-operated calcium channels, are responsible for its activation [5]. The dependent pathway is crucial for many body functions, such as the immune system and cardiac development [6]. Dysregulation of this pathway can lead to diseases, such as autoimmunity or cancer [7]. It synthesizes cyclooxygenase-2 (COX-2), which promotes endothelial cell proliferation and migration [8], which in turn can be blocked pharmacologically by cyclosporin A (CsA), a calcineurin inhibitor [9]. Endogenous calcineurin regulator proteins, such as regulator of calcineurin (RCAN) families, consist of three genes: RCAN1, RCAN2, and RCAN3 [10]. The most extensively studied is RCAN1, which is an endogenous inhibitor of the calcineurin-NFAT pathway. RCAN1 has two isoforms, RCAN1.1 and RCAN1.4, both of which are involved in angiogenesis [11]. From a pathophysiologic standpoint, it is an important player in regulating cardiac hypertrophy, exocrine pancreatic growth, and proper islet beta-cell function.

## Review

Role of NFAT proteins and recent updates

The study of pathway regulation is a vibrant and continuously developing area where each discovery plays a crucial role in enhancing the comprehension of their functions in physiological and pathological contexts. This section offers a comprehensive summary of NFAT proteins. The summary includes the recent studies (2018-2024) on the five members of the NFAT family and their roles in various biological processes. The review is to keep the researchers up to date on the latest developments in the field.

NFATc1

NFATc1 participates in many biological activities, including angiogenesis. Thus, inhibition of NFATc1 pro-angiogenicity in human retinal microvascular endothelial cells could lead to promising treatment targets for ocular diseases [12]. NFATc1 is also related to endothelial-mesenchymal transdifferentiation (EMT), a process implicated in cancer metastasis and lymphangiogenesis [13]. Recently, NFATc1 involvement in osteoclastogenesis and bone metabolism by binding to its specific binding site of the RANK gene promoter has been discovered [14], where RCAN1.4 is shown to suppress osteosarcoma [15]. Since NFATc1 is involved in mammary epithelial morphogenesis [16], inhibiting NFATc1 reduced basal-like breast cancer cells' aggressiveness and therapy resistance [17]. NFATc1 also plays a role in the pathogenesis of Kawasaki disease [18], cervical cancer [19], and glioblastoma [20].

NFATc2

There is a wide range of cancers associated with the dysregulation of NFATc2, such as colon cancer [21], pancreatic carcinoma [22], glioblastoma [23], and colorectal carcinoma [24]. Recent findings also emphasize the relationship between this protein and many types of cancers. Interaction between TRPM7 and NFATc2 has been associated with chemotherapeutic resistance and metastasis in head and neck squamous cell carcinoma [25]. There is a crosstalk between NFATc2, NFKB1/RELA, and the core-regulatory element (CRE) in the ETS1 promoter region in metastatic breast cancer that regulates ETS1 gene transcription [26]. Thus, inhibiting this interaction could be an excellent strategy to reduce the tumor invasiveness of breast cancer cells. In gastric cancer (GC), the transient receptor potential canonical 3 (TRPC3) promotes tumorigenesis via the CNB2/GSK3β/NFATc2 signaling pathway; thus, TRPC3 could be a possible target for therapeutic intervention [27]. It has been found that NFATc2 is regulated by CD147 and matrix metalloproteinase in primary and metastatic melanoma [28]. Two NFATc2-related axes control cancer progression: the NEDD4/FBP1 axis in cholangiocarcinoma [29] and the MYC/NFATc2 axis in acute myeloid leukemia (AML) cells [30]. The protein is also associated with the WNT pathway in non-small cell lung carcinoma [31].

NFATc3

Based on the number of articles, the most extensively studied NFAT protein is NFATc3. Recent studies highlighted the role of NFATc3 in stem cells, human embryonic stem cells [32], and neural stem cells [33]. NFATc3 is also associated with sepsis-induced acute lung injury and pulmonary edema [34]. It has also been shown to be involved in cardiac hypertrophy by promoting mitochondrial fragmentation through the NFATc3/miR-153-3p pathway [35]. For cancer, there are various types of cancer associated with the protein, such as the effect of NFATc3 on hepatitis B virus (HBV) replication and hepatocarcinogenesis, making it a promising target for hepatitis B-induced hepatocellular carcinoma [36]. In ulcerative colitis-associated colorectal cancer, NFATc3 regulates macrophage inflammation and carcinogenesis, mediated by Pou3f1 [37]. NFATC3/PLA2G15 also promotes the invasion and proliferation of colorectal cancer [38]. Retinoic acid-induced 14 (RAI14) facilitated cell proliferation and invasion of GC is regulated by the circNFATC3/miR-23b-3p axis [39]. Recent findings of other cancers associated with this protein are oral squamous cell carcinoma (OSCC) [40,41], pancreatic ductal adenocarcinoma (PDAC) [42], and glioblastoma [43], highlighting its role in cancer progression.

NAFTc4

NFATc4 is less studied compared to the other proteins. Recent updates showed that this protein plays a role in ventilator-induced lung inflammation [44]. NFATc4 is a critical factor in many cancers, such as the AML prognosis [45]. It has also been associated with quiescence and chemotherapy resistance of ovarian cancer [46]. Its inhibition has been shown to suppress melanoma cell migration and invasion [47]. The protein is also involved in metastatic renal cell carcinoma [48]. It also promotes nasopharyngeal carcinoma when activated through transient receptor potential vanilloid type 4 (TRPV4) [49]. The reports implied that NFATc4 is as essential as other NFAT proteins in its role in diseases, particularly cancer.

NFAT5

Previous studies found that NFAT5 is a promising target for coxsackievirus B3 infection, a major cause of viral myocarditis [50]. This might be associated with NFAT being an endogenous arteriogenesis regulator [51]. Targeting this regulatory protein may be a novel strategy for treating ischemic diseases. However, like the other NFAT proteins, NFAT5 also regulates cancer [52,53]. Recent reports also associated the protein with the progression of several other cancers, such as OSCC [54], adrenocortical carcinoma [55], endometrial cancer [56], hepatocellular carcinogenesis [57], and pancreatic cancer [58]. More research is needed, however, to understand the NFAT5 mechanisms fully (Table 1).

**Table 1:** Update on NFAT proteins and their physiological and pathological relationships (2018-2024). NFAT: nuclear factor of activated T cells; CRE: core-regulatory element; FTs: fusion transcripts; LDHA: lactate dehydrogenase A; TNF-α: tumor necrosis factor-alpha; IL-1: interleukin-1; EGFR: epidermal growth factor receptor; NF-κB: nuclear factor-kappa B.

No.	Protein	Physiological/pathological condition	Summary	References
1	NFATc1	Osteoclastogenesis	RANK-NFATc1 signaling promotes osteoclastic differentiation.	Kitazawa et al. (2021) [14]
		Osteosarcoma	RCAN1.4 deficiency promoted cancer cell proliferation, invasion, and migration in vitro.	Huang et al. (2021) [15]
		Mammary epithelial morphogenesis	NFATc1 is a regulatory factor of mammary basal stem/progenitor cells.	McNeil et al. (2021) [16]
		Breast cancer	NFATc1 is upregulated upon loss of f polycomb repressive complex 2 (PRC2) activity. NFATc1 inhibition reduces epithelial-to-mesenchymal transition, aggressiveness, and therapy resistance.	Mieczkowska et al. (2021) [17]
		Kawasaki disease	Ca2+/NFATc1 modulates the pathogenesis of Kawasaki disease vasculitis.	Wang et al. (2020) [18]
		Cervical cancer	NFATc1 regulates IL-10 secretion through the c-myc/PKM2 pathway.	Tan et al. (2022) [19]
		Glioblastoma	DYRK1A increases NFATC1 transcriptional activity.	Liu et al. (2021) [20]
2	NFATc2	Head and neck squamous cell carcinoma	NFAT pathway induces TRPM7 that mediates metastasis and chemotherapeutic resistance.	Chen et al. (2022) [25]
		Breast cancer	ETS1 transcription is regulated by crosstalk between NFATc2, NFKB1/RELA, and CRE region.	Kim et al. (2018) [26]
		Gastric cancer (GC)	Inhibition of the TRPC3-ROCE-CNB2/GSK3-NFATc2 signaling cascade limits tumorigenicity and promotes cell apoptosis.	Lin et al. (2021) [27]
		Melanoma	CD147 affects MMP-9 expression by regulating NFAT1 activity.	Liu et al. (2020) [28]
		Cholangiocarcinoma	NFATc2 is involved in the cholangiocarcinoma progression via the NEDD4/FBP1 axis.	Zhao et al. (2023) [29]
		Acute myeloid leukemia (AML)	NFATc2 and MYC are involved in the maintenance of THP-1 cell function, a potential therapeutic target.	Patterson et al. (2024) [30]
		Non‑small cell lung carcinoma	mRNA expression of NFATc2 could be a diagnostic or prognostic biomarker.	Motamediyan et al. (2023) [31]
3	NFATc3	Human embryonic stem cell	Calcineurin A gamma and NFATc3 contribute to human embryonic stem cell differentiation, through the SRPX2‐uPAR signaling pathway.	Chen et al. (2021) [32]
		Neural stem cells	NFATc3 maintains the stemness of neural stem cells through the regulation of cyclin D1 and p21.	Cao et al. (2021) [33]
		Acute lung injury and pulmonary edema	Inhibition of this protein reduced the sepsis effect.	Karpurapu et al. (2018) [34]
		Cardiac hypertrophy	NFATc3, miR-153-3p, and MFN1 cause a mitochondrial dynamic imbalance in cardiomyocyte hypertrophy.	Wang et al. (2020) [35]
		Hepatocellular carcinoma	NFATc3 binds to the promoter regions of IFNL1 and IFNB1 to activate their transcription in a synergistic manner with the RIG-I pathway, which can inhibit hepatitis B virus replication and hepatocellular carcinoma tumorigenesis.	Zao et al. (2021) [36]
		Colorectal cancer	Pou3f1 facilitates NFATc3 in ulcerative colitis-associated colorectal cancer development.	Lin et al. (2022) [37]
		Colorectal cancer	NFATC3–PLA2G15 FTs promote tumor invasion.	Jang et al. (2019) [38]
		GC	circNFATC3/miR23b-3p axis regulates RAI14, cause cell growth and invasion.	Yan et al. (2021) [39]
		Oral squamous cell carcinoma (OSCC)	circNFATC3 advanced OSCC by regulating the miR-520h/LDHA axis.	Xie et al. (2023) [40]
		OSCC	NFATc3 is essential in maintaining cancer stemness and OSCC progression via the novel NFATc3-OCT4 axis.	Lee et al. (2019) [41]
		Pancreatic ductal adenocarcinoma (PDAC)	SENP3-mediated deSUMOylation modulates NFATc3, which is involved in PDAC tumor progression in hypoxic conditions.	Tong et al. (2022) [42]
		Glioblastoma	NFATc3 controls proliferation and migration capacities.	Urso et al. (2019) [43]
4	NFATc4	Ventilator-induced lung injury	NFATc4 signaling pathway is associated with the release of ICAM-1, VCAM-1, TNF-a, and IL-1, which induces inflammation.	Li et al. (2020) [44]
		AML	NFATc4 is a key gene in AML's poor prognosis through Tregs recruitment.	Zhao et al. (2020) [45]
		Ovarian cancer	NFATc4 is a mediator of quiescence and chemoresistance.	Cole et al. (2020) [46]
		Melanoma	Inhibition of NFATc4 may be effective in melanoma treatment.	Xiao et al. (2020) [47]
		Renal cell carcinoma	CD40 ligation induces the activation of NFATc factors, particularly NFATc4.	Pontrelli et al. (2021) [48]
		Nasopharyngeal carcinoma	TRPV4 promotes the cancer malignant potential through NFAT4 signaling.	Zhang et al. (2022) [49]
5	NFAT5	Arteriogenesis	NFAT5 promotes arteriogenesis and angiogenesis via MCP-1-dependent monocyte recruitment.	Lin et al. (2020) [51]
		OSCC	Under hyperosmotic conditions, NFAT5 activates DPAGT1 and EGFR glycosylation to promote OSCC	Yoshimoto et al. (2021) [54]
		Adrenocortical carcinoma	NFAT5 overexpression and osmotic stress response-related genes indicate its role in adrenocortical tumorigenesis.	Brown et al. (2020) [55]
		Endometrial cancer	NFAT5-HIF-1α-Cox-2 axis is involved in its progression.	Okumura et al. (2024) [56]
		Hepatocellular carcinoma	The protein promotes stemness and cisplatin resistance via ATM-NF-κB.	Lee et al. (2020) [57]
		PDAC	NFAT5 facilitates pancreatic ductal adenocarcinoma cell survival by contributing to the Warburg effect through the transcription of PGK1.	Jiang et al. (2019) [58]

Future outlook of NFAT proteins

This review aimed to demonstrate the steadily expanding research findings associating NFAT proteins with physiological and pathological activities, especially those related to angiogenesis. We have been compiling the most recent research on NFAT proteins because there has been interest in understanding the basic science of the workings of NFAT signaling pathways and the clinical/translational implications of NFAT pathway dysfunction.

Based on the reports highlighted in this review, many gaps can be identified by focusing on the proteins and the pro-angiogenic effect. An example is the relationship between NFATc1 human retinal microvascular endothelial cells [20], which suggests a significant implication in post-cataract surgery management, sparking interest in potential new approaches to post-surgery care. Since excessive vasculogenesis can be detrimental to the cornea, and its suppression may be necessary in treatment. However, inhibiting angiogenesis could slow the healing process, potentially hindering recovery. Therefore, the identification of optimal medication that does not halt the process of angiogenesis underscores the urgent need for studying NFATc1 activity and inhibition.

The second potential avenue of exploration is the role of NFAT proteins in illnesses caused by the immune system, the prevalence of which has increased in recent decades due to lifestyle changes. As discussed in previous sections, NFATc3 and NFATc4 have been linked to lung injury [35,48]. Understanding the roles of NFATc3 and NFATc4 could open up new possibilities for exploring and potentially treating these illnesses, offering hope in the battle against immune system-related diseases.

The most important aspect of NFAT proteins is their relation to angiogenesis in the setting of cancer. While both are required for physiological growth, unfortunately, tumors also utilize them to promote cancer progression, where NFAT dysregulation may be to blame. As mentioned in the previous section, all NFAT proteins initiate and progress many types of cancers. Recent reports also suggest that NFATc4 and NFAT5, which have not previously been extensively associated with cancer, showed otherwise [49-52,55-58]. A critical consequence of these findings is the promising potential for comprehensive research on all NFAT proteins that could identify potential mechanisms for treatment, underlining the importance of this area of study. However, it is also essential to note that the use of drugs for pathway inhibition in chemotherapy would typically result in side effects. To overcome this, new technologies could be used to identify potential drugs, such as computer-aided drug discovery to simulate interactions between the drug and NFAT proteins. Other methods are finding a specific miRNA-based gene for therapy and developing new drug carriers that can precisely deliver inhibitors to the affected tissue. Another possibility is to find a way to administrate local NFAT pathway inhibitors in vivo, for instance in bladder cancer (intravesicular) or pancreatic cancer (intraductal).

## Conclusions

While NFAT proteins participate in various cellular processes, their potential role in angiogenesis is particularly intriguing. Recent discoveries, such as NFATc1 pro-angiogenic effect in retinal microvascular endothelial cells, the association of NFATc3 and NFATc4 with immune-related diseases, and the significant contributions of NFAT proteins to cancer progression, highlight the importance of these molecules and pathways. In summary, continued research into the NFAT pathway, particularly its role in angiogenesis, offers exciting potential for new therapeutic approaches and a deeper understanding of its interactions with other signaling pathways.
